# Recurrent SARS-CoV-2 RNA positivity after COVID-19: a systematic review and meta-analysis

**DOI:** 10.1038/s41598-020-77739-y

**Published:** 2020-11-26

**Authors:** Mahalul Azam, Rina Sulistiana, Martha Ratnawati, Arulita Ika Fibriana, Udin Bahrudin, Dian Widyaningrum, Syed Mohamed Aljunid

**Affiliations:** 1 Department of Public Health, Faculty of Sports Science, Universitas Negeri Semarang, Semarang, 50229 Indonesia; 2 Department of Pulmonology Medicine, SMC Telogorejo Hospital, Semarang, Indonesia; 3 Department of Cardiology and Vascular Medicine, Faculty of Medicine, Universitas Diponegoro, Semarang, Indonesia; 4 Department of Clinical Pathology, Dr. Kariadi Hospital, Semarang, Indonesia; 5 Department of Health Policy and Management, Faculty of Public Health, Kuwait University, Kuwait, Kuwait

**Keywords:** Infectious diseases, SARS-CoV-2, SARS virus, Viral epidemiology

## Abstract

Present study aimed to estimate the incidence of recurrent SARS-CoV-2 RNA positivity after recovery from COVID-19 and to determine the factors associated with recurrent positivity. We searched the PubMed, MedRxiv, BioRxiv, the Cochrane Library, ClinicalTrials.gov, and the World Health Organization International Clinical Trials Registry for studies published to June 12, 2020. Studies were reviewed to determine the risk of bias. A random-effects model was used to pool results. Heterogeneity was assessed using *I*^2^. Fourteen studies of 2568 individuals were included. The incidence of recurrent SARS-CoV-2 positivity was 14.8% (95% confidence interval [CI] 11.44–18.19%). The pooled estimate of the interval from disease onset to recurrence was 35.4 days (95% CI 32.65–38.24 days), and from the last negative to the recurrent positive result was 9.8 days (95% CI 7.31–12.22 days). Patients with younger age and a longer initial illness were more likely to experience recurrent SARS-CoV-2 positivity, while patients with diabetes, severe disease, and a low lymphocyte count were less likely to experience. Present study concluded that the incidence of recurrent SARS-CoV-2 positivity was 14.8% suggesting further studies must be conducted to elucidate the possibility of infectious individuals with prolonged or recurrent RNA positivity.

## Introduction

Globally, the reported number of confirmed infections and deaths due to the severe acute respiratory syndrome coronavirus 2 (SARS-CoV-2) pandemic was 7,410,510 and 418,294, respectively, by June 12, 2020^1^. Country governments have implemented public health measures such as lockdowns, physical distancing, use of face masks, and frequent hand-washing; however, the incidence of SARS-CoV-2 infection is still increasing. The proportion of severe cases and case fatality rates have been reported to be 25.6% and 3.6%, respectively^2^, with individuals with comorbidities being at greater risk of developing severe disease^2,3^.

The World Health Organization (WHO) has provided criteria for assessing the recovery of patients hospitalized with coronavirus disease 2019 (COVID-19), i.e. generally after clinical recovery and two negative PCR swabs > 24 h apart^4^. Recently, there have been several reports of recurrent SARS-CoV-2 RNA positivity in individuals who had recovered from COVID-19^5,6^. with estimates of the incidence of recurrent SARS-CoV-2 positivity in individuals who have recovered from COVID-19, ranging from 7.3^5^ to 21.4%^6^. However, to date no systematic reviews have been published to provide a pooled estimate of the incidence of recurrent positivity. This systematic review aimed to: estimate the incidence of recurrent SARS-CoV-2 positivity and determine the characteristics and risk factors related to the recurrent SARS-CoV-2 positivity in patients who had recovered from COVID-19.

## Methods

### Protocol and registration

This review is written following the Preferred Reporting Items for Systematic Review, and Meta-Analysis Protocol (PRISMA-P)^7^ (Supplementary Table 1). The protocol of this review was published in the International Prospective Register of Systematic Reviews (PROSPERO) on May 14, 2020, reference no. CRD42020186306^8^.

### Search strategy and information resources

A search was conducted of PubMed, MedRxiv, BioRxiv, Cochrane Library, ClinicalTrials.gov, the WHO international register of clinical trials registry using the search term in Medical Subjects Headings (MeSH) and free text: ("2019 nCoV" OR "2019nCoV" OR "2019 novel coronavirus" OR "COVID 19" OR "COVID19" OR "new coronavirus" OR "novel coronavirus" OR "SARS CoV-2" OR (Wuhan AND coronavirus) OR "COVID 19" OR "SARS-CoV" OR "2019-nCoV" OR "SARS-CoV-2") AND ((recurrence) OR (relapse) OR (re*infection) OR (re*activation)).

### Data management and study selection

Literature search results were organized using Mendeley (Mendeley, Ltd, Elsevier, UK). Article titles and abstracts retrieved from the databases were transferred to Mendeley citation manager after being screened and checked for duplication. All records that did not meet the eligibility criteria were excluded from the review.

The eligibility of articles based on their title and abstract was assessed independently by MA and AF. If necessary, the full paper was retrieved to further determine the eligibility status. In cases of disagreement regarding eligibility, consensus was reached by consulting a third reviewer (MR). The eligibility criteria were: (i) the study designs are cross-sectional, case–control or cohort design; (ii) the study reports the incidence of recurrent SARS-CoV-2 positivity in individuals who had recovered from COVID-19 and its related factors; and (iii) the articles included published or unpublished studies. The published studies may included both peer-reviewed reports and pre-print reports. Studies in languages other than English were excluded if no translated version of the manuscript was available. In this study, we did not consider the terms recurrent SARS-CoV-2 positivity and recurrent SARS-CoV-2 infection synonymous.

The full text of the articles that met the eligibility for the review were then assessed. If the data provided in the articles were incomplete, the author was contacted to obtain complete data. Data collection forms were used for specific purposes, including the screening process, determining eligibility, data collection, and incomplete data identification as well as the risk of bias assessment.

### Data extraction and quality assessment

The following data items were extracted: authors, funding, study design, the population of the study, number of episodes of recurrent SARS-CoV-2 positivity per case, and patient characteristics. The patient characteristics considered included age, sex, body mass index, clinical/laboratory manifestations, and comorbidities such as diabetes and hypertension. The outcome was recurrent SARS-CoV-2 positivity in individuals who had recovered from COVID-19, determined as based on positive result of reverse transcription polymerase chain reaction (RT-PCR) on re-testing, after being followed-up or re-admitted after discharged from hospital.

We used the quality assessment tool for cross-sectional and cohort studies published by the National Institutes of Health^9^ to assess the methodological quality of included studies and the risk of bias as described previously^2^. Each item was scored 0 or 1 point based on the criteria. A total of all items ranged from 0 to 14 was used to assess the quality of the article. Based on the overall score, we categorized articles to high risk of bias with score ≤ 6, medium risk of bias with a score of 7–10, and low risk of bias when the score was ≥ 11. Each study was assessed for risk of bias independently by MA and MR. Any disagreement in the risk of bias assessment was resolved by discussion to reach consensus or by consulting UB and SA.

### Data analysis

We performed data analysis using Revman (Review Manager version 5.3.5 Copenhagen, The Nordic Cochrane Centre, 2014). Random-effects meta-analysis was used to calculate the pooled incidence of recurrent SARS-CoV-2 positivity with 95% confidence intervals. The incidence for each individual study with its standard error (SE) adds to the study data in RevMan. If the SE was not reported and the raw data could not be accessed, the SE was calculated using the formula $SE=\sqrt{(p}(1-p)/n)$. Meta-analysis was used to calculate pooled estimates of the time from disease onset to recurrent test positivity and the time from the last negative test result to recurrent positivity.

Meta-analysis was also used to calculate the pooled relative risk (RR) of recurrent SARS-CoV-2 positivity according to age, sex, hypertension, diabetes, other co-morbidities, disease severity, body mass index (BMI), fever as the initial presenting compliant, days from onset to negative conversion, lymphocyte count, D-dimer, and lung consolidation. We then assessed the heterogeneity between studies using *I*^2^, with values of 25%, 50%, and 75% representing low, moderate, and high heterogeneity, respectively. A sensitivity analysis was also performed for non-peer-reviewed as well as for the PCR test specimens’ type.

## Results

**Figure 1:**
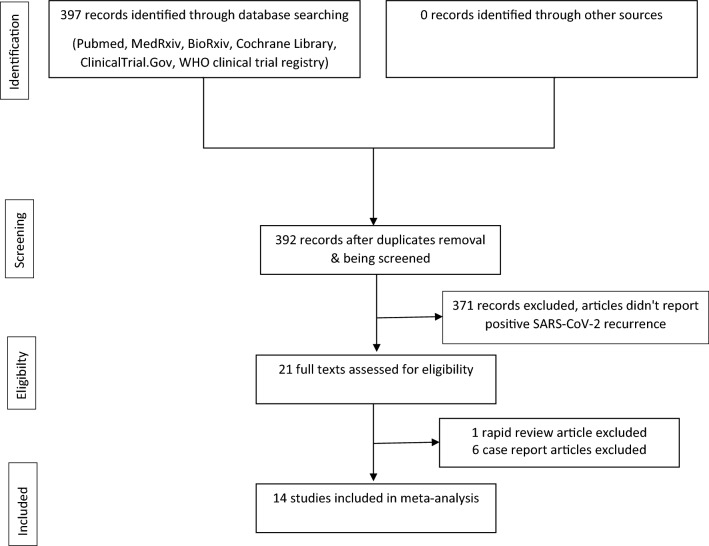
PRISMA-P study selection diagram.

Our search on June 12, 2020, produced 397 records. Of these records, 392 were left after the duplicates were removed. Of these records, 371 were excluded from the review because the articles did not report recurrent SARS-CoV-2 positivity. Of this, 21 full texts were assessed for eligibility and 14 studies were included in the meta-analysis (Fig. 1).

Table 1 summarizes the characteristics of the finally selected studies. These studies were published between Mar 17 and May 29, 2020. We included an article of non-peer-reviewed study. There was a total of 2,568 participants from all the studies combined, of which 318 experienced recurrent SARS-CoV-2 positivity. Thirteen of the 14 studies were conducted in China and one study was conducted in Brunei. Four of the Chinese studies were conducted in the city of Wuhan and the rest were conducted in other cities. There were six studies (43%) with a cross-sectional design, and four studies (29%) each with a retrospective cohort and prospective cohort design. The most frequently used sample types were nasopharyngeal or oropharyngeal swabs only (46%), and the remaining studies used a variety of sample types including fecal, nasopharyngeal, and oropharyngeal swabs. One study did not report the type of sample that was used^10^. The median interval duration from disease onset to recurrence ranged from 21 to 50 days, while the interval from the last negative to recurrent positive result ranged from 4 to 19 days. The risk of bias was assessed as low in seven studies (50%), moderate in six studies (43%), and high in one study (7%) (Supplementary Table 2).

**Table 1:** Study characteristics included in the meta-analysis.

Study	Published date	City	Country	Peer-reviewed published	Study design	Funding	Number of RP	Number of population	Specimens of PCR retest	Onset to RP (days)	Negative to RP (days)
An^11^	30/03/2020	Shenzhen	China	Yes	Cohort	N/A	38	242	Fecal and nasopharyngeal	N/A	Range: 5–7
Chen^12^	12/05/2020	Wuhan	China	Yes	Retrospective cohort	Guanggu Branch of Hubei Province Maternity and Childcare Hospital Fund	81	1067	Oropharyngeal	Median: 50 IQR: 36.5–59.5	Median: 9 IQR: 7–10
Huang ^13^	10/05/2020	Shenzhen	China	No	Cohort	Sanming Project of Medicine in Shenzhen Bill & Melinda Gates Foundations; National Natural Science Foundation of China	69	414	Nasopharyngeal	Median: 37 (N1* = 53) Median: 41 (N2* = 13) Median: 24 (N3* = 3)	Median: 19 Range: 6–52 (N = 69)
Hui Zhu^14^	11/05/2020	Zhejiang	China	Yes	Retrospective cohort	Ningbo HwaMei Key Research Fund and Key Laboratory of Diagnosis and Treatment of Digestive System Tumors of Zhejiang Province	17	98	Nasopharyngeal	Median: 21 IQR: 17–28 (Onset to negative)	Median: 4 IQR: 3–8.5
Jiang^10^	17/03/2020	Shangqiu	China	Yes	Crossectional	None	6	35	N/A	Median: 32.5 IQR: 31.25–36	Median: 10 IQR: 9.25–10
Li^15^	20/04/2020	Zhejiang	China	Yes	Cohort	Zhejiang University special scientific research fund for COVID-19 prevention and control	6	13	Sputum (oro-/naso-pharyngeal), fecal	Median: 32.5 IQR: 30.25–39.25	Median: 10.5 IQR: 6.25–14
Ling^5^	05/05/2020	Shanghai	China	Yes	Retrospective cohort	First-class university and first-class discipline building project of the Fudan University and the Scientific research for special subjects on 2019-NCoV of the Shanghai Public Health Clinical Center	11	66	Fecal	N/A	N/A
Liu^16^	29/05/2020	Wuhan	China	Yes	Crossectional	National Key Research and Development Program of China	11	150	Oropharyngeal	Median: 38 IQR: 35–44	N/A
Wong^17^	05/05/2020		Brunei	Yes	Crossectional	None	21	106	Nasopharyngeal	Median: 32 IQR: 28.75–33.5	Median: 14 IQR: 13.5–16
Wu^18^	22/05/2020	Loudi	China	Yes	Crossectional	Grants No. 81902094 and 81600497 from the National Natural Science Foundation of China (Dr Zhou) and grant No. 2019RS1036 from the Science and Technology Plan Project of Hunan Province (Dr P.Wu)	10	60	Fecal and nasopharyngeal	Median: 21 IQR: 16.5–22.75	Median: 11 IQR: 6.5–17
Xiao^6^	09/04/2020	Wuhan	China	Yes	Crossectional	None	15	70	Oro-/naso-pharyngeal	N/A	N/A
Ye^19^	20/03/2020	Wuhan	China	Yes	Retrospective cohort	Medical Science Advancement Program (Clinical Medicine) of Wuhan University	5	55	oropharyngeal	N/A	Median: 9 IQR: 8–15
Yuan^20^	08/04/2020	Shenzhen	China	Yes	Crossectional	Sanming Project of Medicine in Shenzhen (SZSM201512005)	25	172	Fecal and nasopharyngeal	N/A	Mean: 5.23 ± 4.13 (after discharge)
Zheng^21^	20/04/2020	Whenzou	China	Yes	Cohort	N/A	3	20	Fecal and nasopharyngeal	N/A	7 (after discharge)

*RP* recurrence positive, *PCR = rt-PCR *reverse transcription-polymerase chain reaction.

Onset to negative and Negative to RP: negative determined as last (2nd) negative.

Discharge from hospital, one day after 2nd negative.

N1*, N2*, N3*: patients with 2, 3, and 4 admission, respectively.

**Figure 2:**
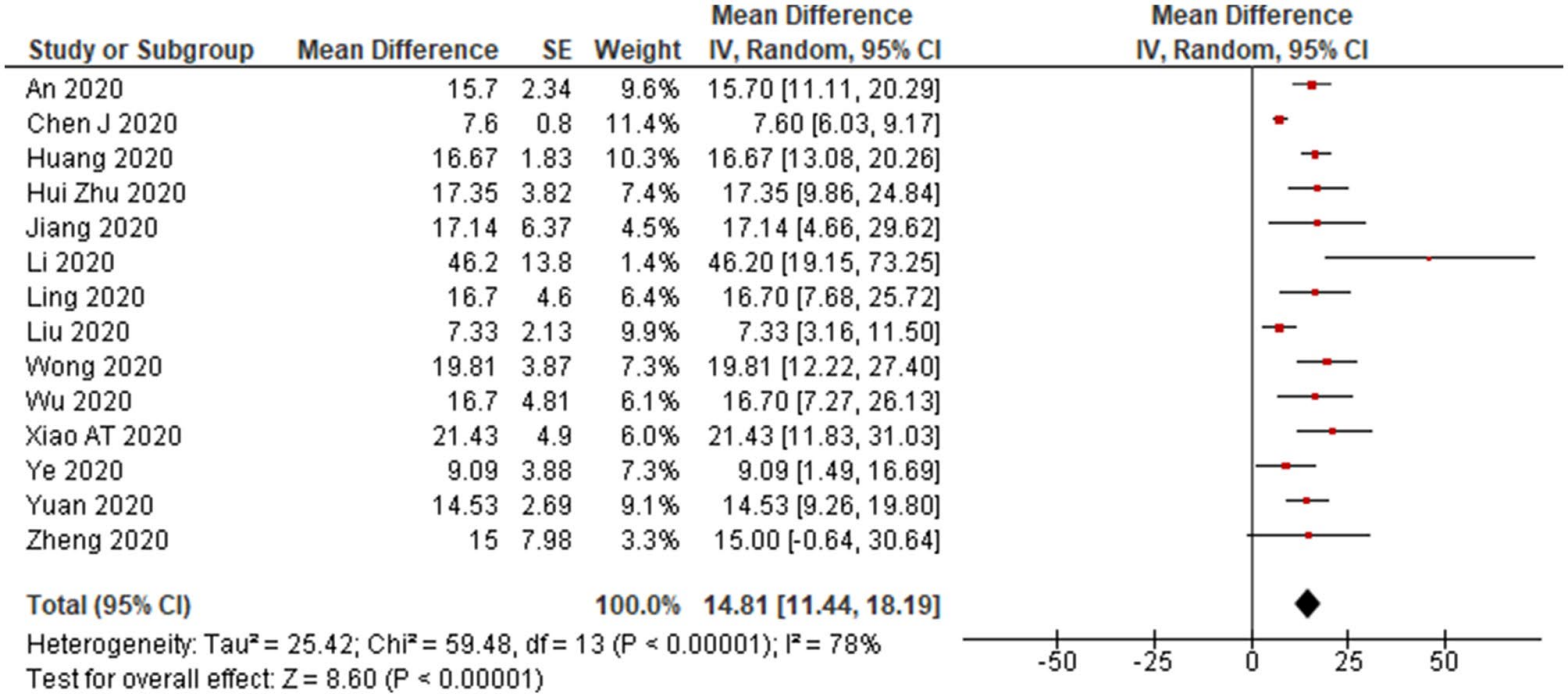
A meta-analysis of the pooled estimated incidence of recurrent SARS-CoV-2 RNA positivity.

The pooled estimate of the incidence of recurrent SARS-CoV-2 positivity was 14.8% (95% CI 11.44–18.19%) (Fig. 2). Liu et al.^16^ found the lowest incidence (7.33%, N = 150), and Li et al.^15^ found the highest incidence (46.2%, N = 13). The pooled incidence in the peer-reviewed only studies was similar to that of the total studies, i.e., 14.6% (95% CI 11.05–18.09%) with *I*^2^ of 75%. We observed differences in the pooled estimate incidence for recurrent SARS-CoV-2 RNA positivity based on specimen type. The highest pooled incidence of recurrent positivity was nasopharyngeal only specimens (17.3%), followed by fecal only (16.7%), combined [(oro-/naso-pharyngeal and fecal) 16.1%], oro-/naso-pharyngeal only (13.5%), and oropharyngeal only (7.6%). (Supplementary Table 3).

**Figure 3:**
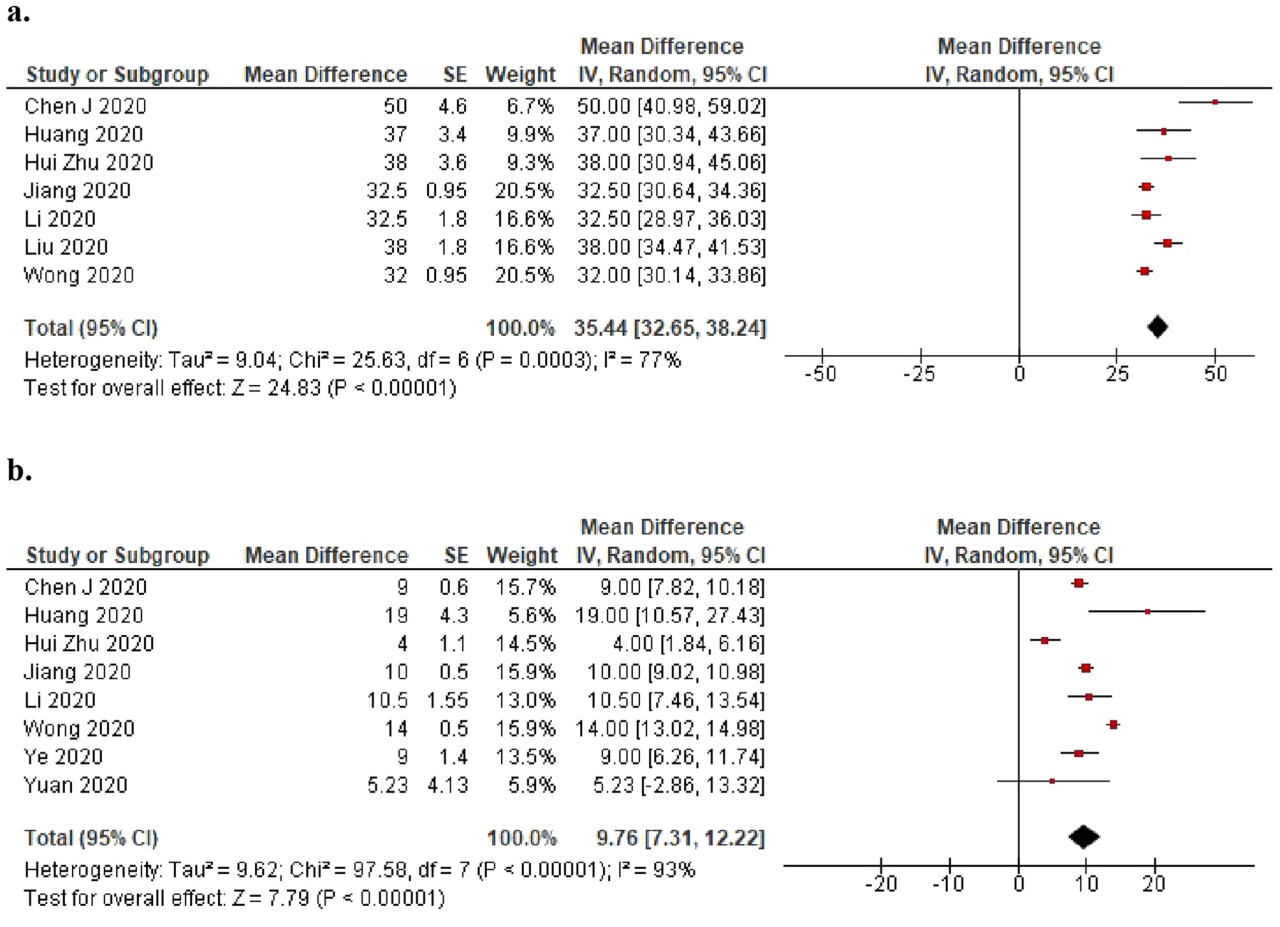
(**a**) A meta-analysis of the pooled estimated interval from onset to recurrent SARS-CoV-2 RNA positivity (days) and (**b**) A meta-analysis of the pooled estimated interval from last negative to recurrent SARS-CoV-2 RNA positivity (days).

Seven studies provided results on the time from disease onset to recurrent positivity, and eight studies provided results on the time from testing negative to recurrent positivity. The pooled estimate of the interval from disease onset to recurrent positivity was 35.4 days (95% CI 32.65–38.24 days), and the pooled estimate of the last negative to recurrent positivity was 9.8 days (95% CI 7.31–12.22 days) (Fig. 3a,b).

**Figure 4:**
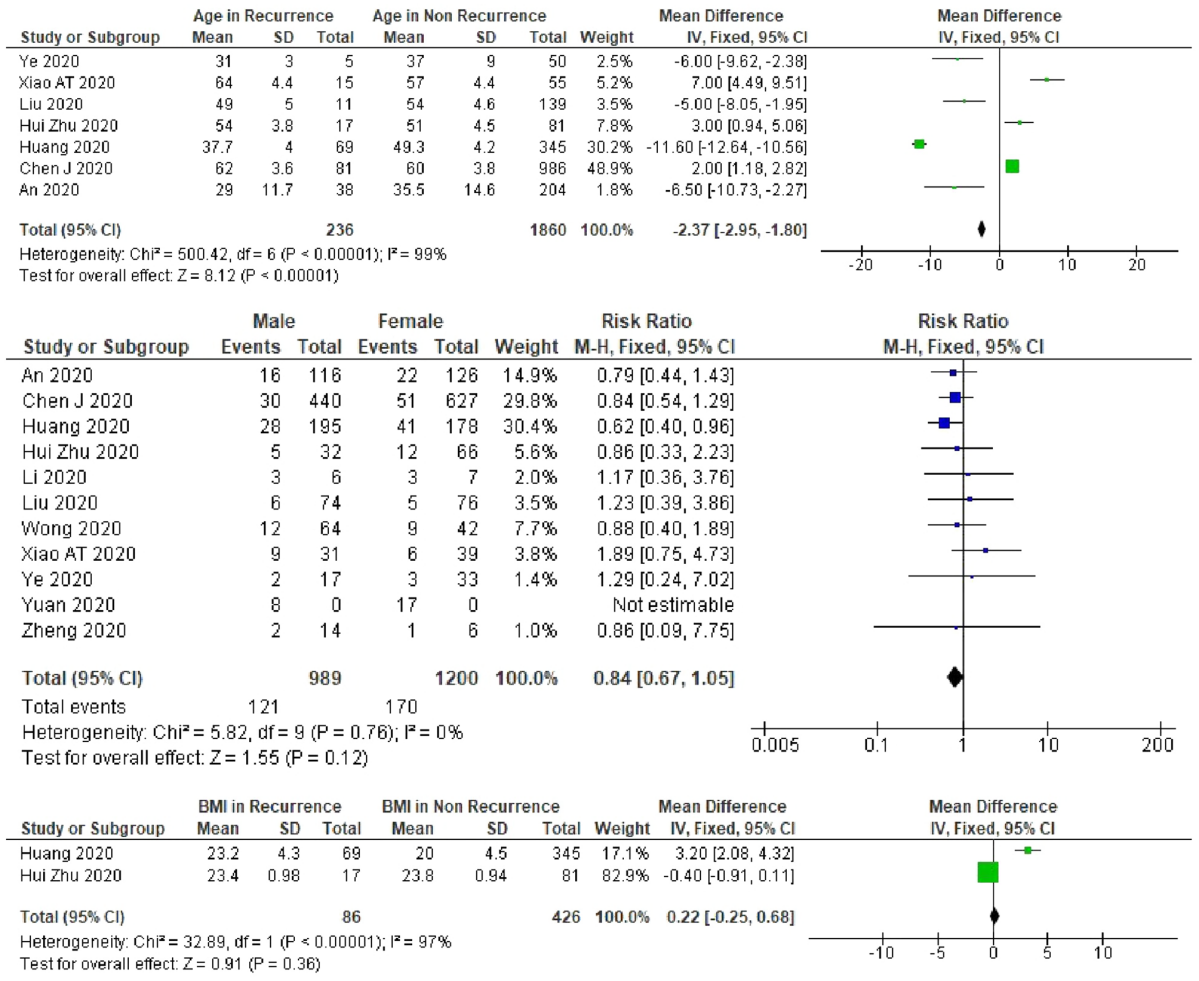
A meta-analysis of the pooled estimated RR of age, sex, and BMI to recurrent SARS-CoV-2 RNA positivity.

Patients with younger age were more likely to experience recurrent SARS-CoV-2 positivity (mean difference: − 2.4, 95% CI − 2.95 to − 1.80), but there was considerable heterogeneity between studies in the effect of age (*I*^2^ = 99%) (Fig. 4). The results of our meta-analysis for sensitivity of the nasopharyngeal only specimens, oro-/naso-pharyngeal only specimens, or combined specimens (oro-/naso-pharyngeal and fecal specimens) were consistent with that of the total studies. Contrariwise, after excluding the non-peer-reviewed study by Huang^13^, which had the largest sample size of nasopharyngeal only specimens in this systematic review, we did not observe any association between age and recurrent SARS-CoV-2 positivity (Table 1 and Supplementary Table 4).

**Figure 5:**
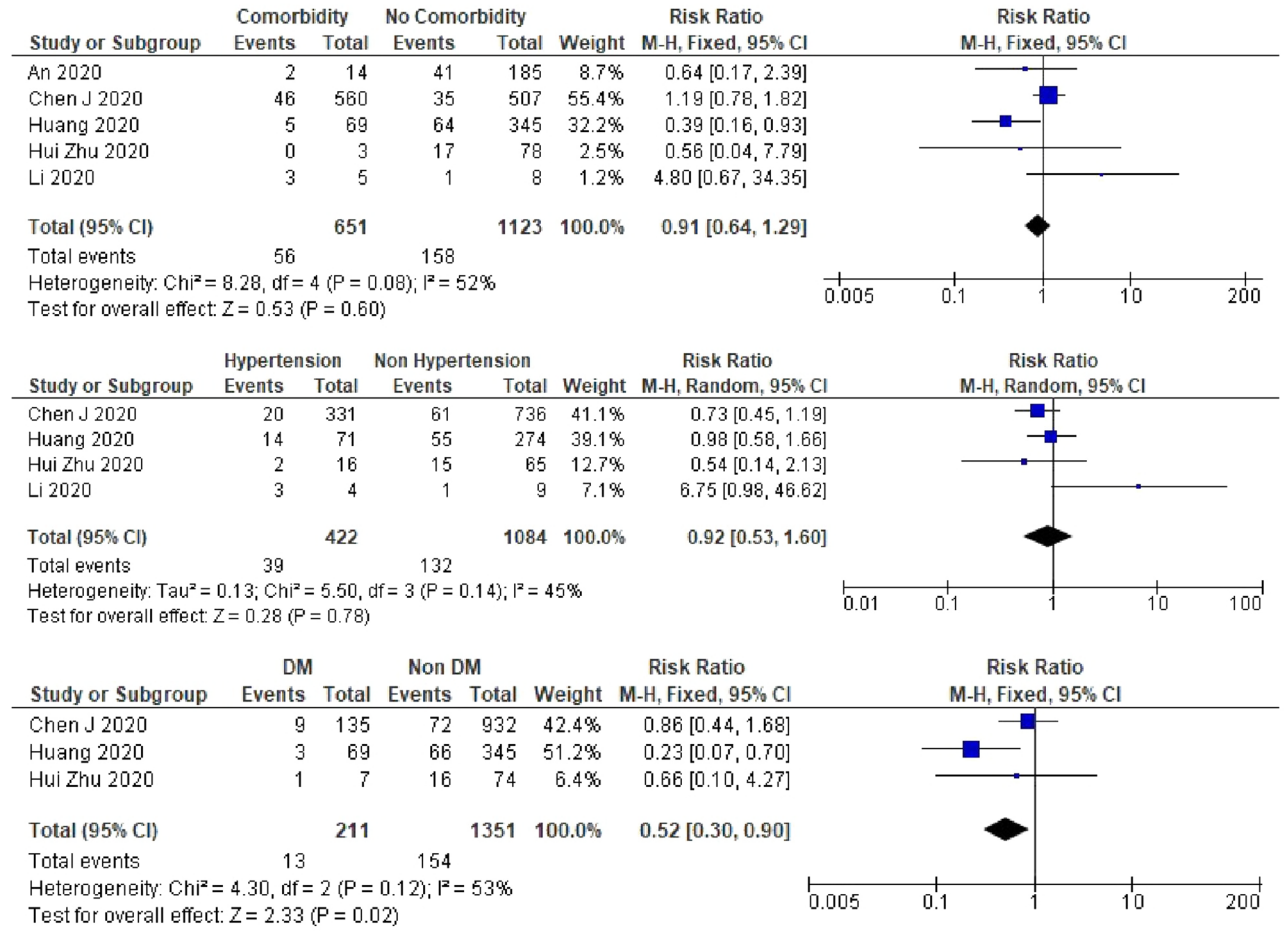
A meta-analysis of the pooled estimated RR of comorbidity, hypertension, and DM to recurrent SARS-CoV-2 RNA positivity.

Patients with diabetes were less likely to experience recurrent SARS-CoV-2 positivity (RR 0.5, 95% CI 0.30–0.90, *I*^2^ = 53%) (Fig. 5). In all the studies which included patients with diabetes PCR tests were performed on oro-/naso-pharyngeal only specimens. Pooled RR was 0.3 (95% CI 0.11–0.72) in the nasopharyngeal only, which was lower than that of the total studies [RR 0.5 (95% CI 0.30–0.90)]**.** However, the meta-analysis results for sensitivity analysis after excluding the non-peer-reviewed study^13^ found no relationship between diabetes and recurrent SARS-CoV-2 positivity (Supplementary Table 5).

**Figure 6:**
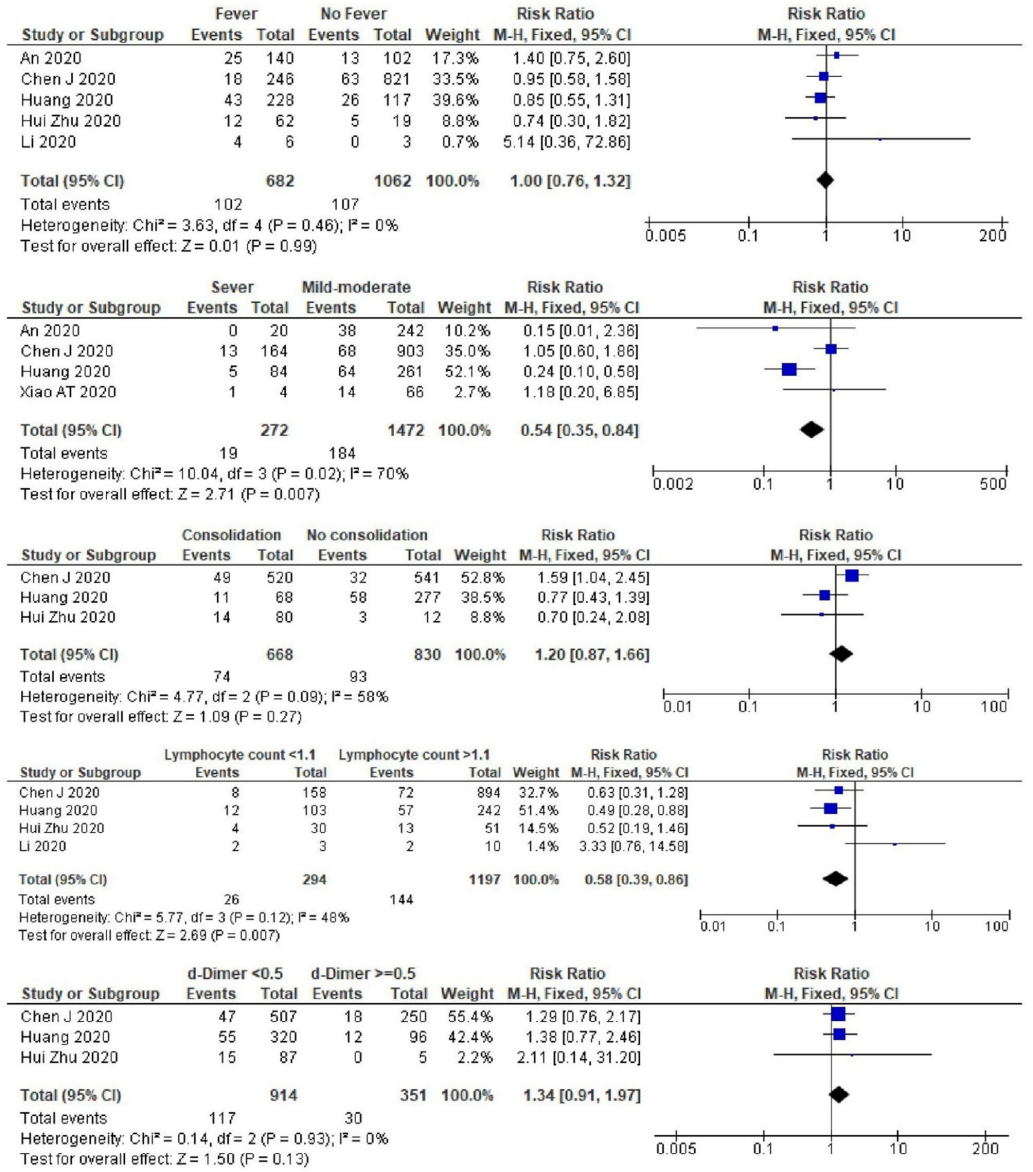
A meta-analysis of the pooled estimated RR of fever and clinical features to recurrent SARS-CoV-2 RNA positivity.

**Figure 7:**
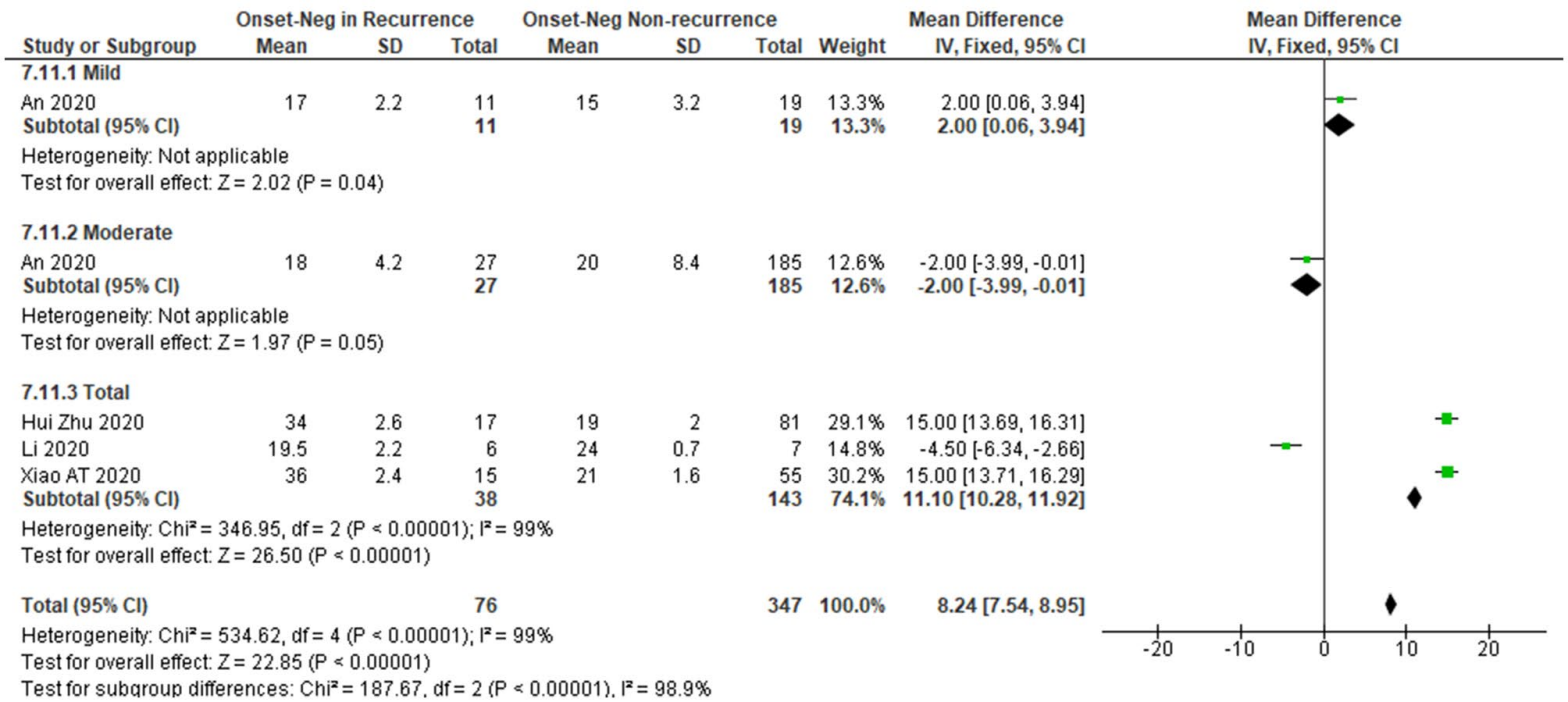
A sub-group meta-analysis of severity for the pooled estimated interval from onset to last negative to recurrent SARS-CoV-2 RNA positivity.

Patients with severe COVID-19 were also less likely to experience recurrently positivity than those with less severe disease (RR 0.5, 95% CI 0.35–0.84, *I*^2^ = 70%) (Fig. 6). Most studies which analyzed the severity status of the disease included oro-/naso-pharyngeal only PCR test specimens. The pooled estimated RR was lowest in nasopharyngeal only study (RR 0.5), followed by total studies and oro-/naso-pharyngeal only studies with pooled RR of 0.5 and 0.6, respectively. We also did not observe any relationship between severity and recurrent positivity after excluding the non-peer-reviewed study by Huang^13^ (Supplementary Table 5). A longer interval from disease onset to the last negative PCR result during the first admission was associated with a greater risk of recurrent SARS-CoV-2 positivity (mean difference: 8.2 days, 95% CI 7.54–8.95 days, *I*^2^ = 98.9%) (Fig. 7).

Patients with a low lymphocyte count (< 1.1 × 10^9^/L) had a lower risk of experiencing recurrent SARS-CoV-2 positivity (RR 0.6, 95% CI 0.39–0.86, *I*^2^ = 48%) (Fig. 6). A meta-analysis result of the lymphocyte count in all studies was similar to the result in oro-/naso-pharyngeal only PCR test specimens. In the peer-reviewed only studies, after excluding the study by Huang^13^, we did not find any relation between lymphocyte count and recurrent positivity (Supplementary Table 6). We did not find an association between sex, BMI, co-morbidity, hypertension, fever, lung consolidation, or D-dimer and the risk of recurrent SARS-CoV-2 positivity (Figs. 4, 5, 6). However, in studies which included nasopharyngeal only specimens, we found that patients with comorbidity were less likely to experience recurrent positivity (RR 0.4, 95% CI 0.18–0.92, *I*^2^ = 0%) (Supplementary Table 6). Furthermore, in these studies, male patients were also less likely to experience recurrent positivity (RR 0.5, 95% CI 0.49–0.99, *I*^2^ = 0%) (Supplementary Table 4).

## Discussion

We conducted a systematic review and meta-analysis of 14 studies involving 2568 individuals. This is the first systematic review on recurrent SARS-CoV-2 RNA positivity among individuals who have recovered from COVID-19. The pooled estimate of the incidence of recurrent SARS-CoV-2 positivity was 14.8%, confirming that recurrent positivity among patients who have recovered and been discharged from hospital is relatively common. The persistence of SARS-CoV-2 protein in some patients with recurrent SARS-CoV-2 positivity may be a sign of active viral replication and so these patients could still be infectious, although the level of infectiousness of individuals with recurrent positivity requires further evaluation. No studies in this review provided evidence of new infections in the family members or close contacts of the recovered patients that experienced recurrent positivity. Several studies clearly reported that there was no new infection infected from the patients with recurrent positivity, the study reported by Lan et al.^22^ found that there were no family members infected. However, these results do not rule out the possibility that individuals with repeat positivity may still be infectious because most patients are likely to have strictly obeyed self-isolation protocols, as described by Zheng et al.^21^.

The highest pooled estimated recurrent positivity incidence was from the studies with nasopharyngeal only specimens (17.3%), while the lowest was in studies with oropharyngeal only specimens (7.6%). The PCR test specimens probably influence the detection rate of recurrent positivity. As previously reported in a systematic review, the positive rate (PR) for nasopharyngeal swab, fecal, and oropharyngeal swab were 45.5%, 32.8%, and 7.6%, respectively^23^, with the highest reported detection rate being from specimens collected from the bronchoalveolar lavage fluid (PR of 92%)^23^. None of the studies included in our meta-analysis had collected samples from bronchoalveolar lavage fluid. We presume that combined sampling site for specimens increases detection of RNA positivity retesting when individually had a high detection rate and decreases detection when individually had a low detection rate. In this systematic review, the combined sampling used oro- or naso- pharyngeal swab and fecal specimens with the pooled incidence of 16.1% as shown in the (Supplementary Table 3).

We estimated that the interval between the onset of the initial episode of the disease and recurrent positivity was 35.4 days. The longest interval (50 days) was reported by Chen^12^. The time from the last negative PCR test result (used as a discharge criterion) to recurrent positivity was 9.8 days, with the longest interval (19 days) being reported by Huang^13^. Regarding the incubation period, Jing et al.^24^ reported that the estimated median of incubation period was 8.1 days and the 99th percentile was 20.6 days. Considering these findings, further studies should be conducted to elucidate whether prolonged persistent and recurrent RNA positivity remain potentially infectious.

Prolonged viral shedding could be considered as the underlying mechanism of recurrent positivity as false-negative PCR test results have been reported^25–29^. The estimated duration of viral shedding based on the absence of SARS-CoV-2 RNA detection was 20 days^30^. However, the presence of nucleic acid alone cannot be used to determine whether viral shedding occurred or potential infectiousness. Viral RNA could still be detected in a long time after the disappearance of active virus^31^, and Yan et al.^32^ categorized prolonged viral shedding with the cut-off of 23 days.

Our review found that younger age, a longer length of stay during the initial illness, and higher lymphocyte count was associated with an increased risk of recurrent positivity, while the presence of diabetes mellitus, severe clinical feature were associated with a reduced risk.

Several studies have reported the determinants of prolonged viral shedding. A systematic review^33^ concluded that the use of corticosteroid was associated with delayed viral clearing. Another review^34^ reported that clearance of SARS-CoV-2 took longer in patients with gastrointestinal disease than in those with respiratory disease, especially in children. A case series reported by Huang et al. found that fecal specimens tended to have persistently detectable SARS-CoV-2 on molecular tests for longer than other specimen types^35^. Another study^32^ reported that in adult patients, especially older patients had prolonged viral shedding, and that treatment with lopinavir/ritonavir was associated with a shorter shedding period. Prolonged viral shedding has also been reported to be associated with male sex, old age, concomitant hypertension, delayed admission to hospital after illness onset, severe illness at admission, invasive mechanical ventilation, and corticosteroid treatment^36^.

The present review showed that in patients with diabetes, elderly patients, and those with severe clinical features, recurrent SARS-CoV-2 positivity was less likely. Previous studies have revealed that diabetes and severe clinical features increase mortality^37^, while older age led to slower recovery^38^. Further study should elucidate the outcome of COVID-19 in older patients with multiple comorbidities, which may influence the lower recovery or negative SARS-CoV-2 RNA status upon hospital discharge.

This review did not consider the underlying mechanism of the recurrent SARS-CoV-2 positivity; however, a previous non-systematic review^39^ assessed possible mechanisms underlying recurrent SARS-CoV-2 positivity and was unable to determine whether it was attributable to false-negative results, reactivation, relapse or reinfection.

A study by Bao et al. give evidence that suggested that reinfection was unlikely. They conducted trials on Rhesus macaques that were re-infected with SARS-CoV-2 on the early recovery phase from initial infection characterized by weight loss, interstitial pneumonia, and systemic viral dissemination mainly in respiratory and gastrointestinal tracts. The results showed that primary SARS-CoV-2 infection protects from subsequent reinfection^40^. Wang et al. also reported that there is no infectious risk of COVID-19 patients with long-term fecal SARS-CoV-2 RNA positivity, and that there were no abnormalities in the gastrointestinal examination of these patients after they had been discharged^41^. However, a case report from Italy by Loconsole et al. described a case of a 48-year-old man with re-detectable positive SARS-CoV-2 after two consecutive negative SARS-CoV-2 molecular tests following his discharge from the hospital. A month after home quarantine, the man developed new symptoms of dyspnea and chest pain, causing him to re-admitted and his SARS-CoV-2 RNA test was positive on his readmission^42^, making it necessary to consider reinfection or recurrence (relapse) as possible mechanisms for recurrent SARS-CoV-2 RNA positivity on retesting. Further studies with larger sample sizes, more longer follow-up, and more detailed measurements should be conducted to determine the mechanisms underlying recurrent positivity.

We performed a sensitivity analysis for non-peer-reviewed study and those with PCR test specimens. We observed the overall meta-analysis results for all the studies were very close to those which included nasopharyngeal only PCR test specimens. The non-peer-reviewed study by Huang^13^ had the second-largest sample size in this review, after the Chen^12^ study. Huang^13^ study used the nasopharyngeal only specimens, while the Chen^12^ study used oropharyngeal only specimens. We believe that the large number of the cohort in Huang^13^ study may have contributed to the results of the meta-analysis and influenced our results. We also realize that our meta-analysis produced large heterogenicity in some parameters reported; however, a sub-group analysis and meta-regression could not be conducted to identify sources of between-study heterogeneity in the pooled incidence estimates, because of insufficient study data. A further review should be re-conducted once additional publications become available, especially by filtering the studies with nasopharyngeal specimens, which have medium rate of detection, or including only studies which have taken the specimens from bronchoalveolar lavage fluid, which has a high rate of detection, in a large cohort.

## Conclusion

This systematic review provides evidence of SARS-CoV-2 recurrence of 14.8% among COVID-19 patients. This review also provides pooled estimated time of onset to the re-detectable positive duration was 35.4 days, and estimated time of the last negative to re-detectable positive duration was 9.8 days. Patients with younger age, no history of diabetes, mild and moderate severity, longer duration of onset to the last negative PCR, and higher lymphocyte count are more likely to experience recurrent SARS-CoV-2 positivity. The current systematic review also showed that PCR test specimens’ type influences the meta-analysis results, suggesting further review should selecting the higher detection rate of specimens’ type. Further studies are also needed to elucidate the possibility of transmission from individuals with prolonged or recurrent RNA positivity.

## Supplementary information

Supplementary Information.

## Data Availability

The datasets analyzed in the current study are available in Figshare repository at https://doi.org/10.6084/m9.figshare.12816410.
