# Supplementary material for: Recurrent SARS-CoV-2 RNA positivity after COVID-19: a systematic review and meta-analysis
Source: Sci Rep. 2020 Nov 26;10:20692. doi: 10.1038/s41598-020-77739-y (PMC7691365; doi:10.1038/s41598-020-77739-y)
Supplement: Supplementary file 1 — Supplementary Information. [file 41598_2020_77739_MOESM1_ESM.pdf]

Recurrent SARS-CoV-2 RNA positivity after COVID-19: A systematic review and meta-analysis

Mahalul Azam, Rina Sulistiana, Martha Ratnawati, Arulita Ika Fibriana, Udin Bahrudin, Dian Widyaningrum, Syed Mohamed Aljunid

Supplementary Table 1. PRISMA-P (Preferred Reporting Items for Systematic review and Meta-Analysis Protocols) 2015 checklist: recommended items to address in a systematic review protocol*

Section and topic	Item No	Checklist item
**ADMINISTRATIVE INFORMATION**		
Title:		
Identification	1a	Stated in title: Recurrent SARS-CoV-2 RNA positivity after COVID-19: A systematic review and meta-analysis
Update	1b	Current study is not an update of a previous systematic review
Registration	2	The protocol of this review was published in the PROSPERO on May 14, 2020, reference no. CRD42020186306
Authors:		
Contact	3a	Mahalul Azam[a,*], Rina Sulistiana[a], Martha Ratnawati[b], Arulita Ika Fibriana[a], Udin Bahrudin[c], Dian Widyaningrum[d], Syed Mohamed Aljunid[e]<br>[a]Department of Public Health, Faculty of Sports Science, Universitas Negeri Semarang, Semarang, Indonesia<br>[b]Department of Pulmonology Medicine, SMC Telogorejo Hospital, Semarang, Indonesia<br>[c]Department of Cardiology and Vascular Medicine, Faculty of Medicine, Universitas Diponegoro, Semarang, Indonesia<br>[d]Department of Clinical Pathology, Dr. Kariadi General Hospital, Semarang, Indonesia<br>[e]Department of Health Policy and Management, Faculty of Public Health, Kuwait University, Kuwait, Kuwait<br>List of authors' emails (listed in athors order):<br>mahalul.azam@mail.unnes.ac.id;rinasulistiana25@gmail.com;martha.bumiwana@gmail.com;arulita.ika.f@mail.unnes.ac.id; bahrudin00@lecturer.undip.ac.id; dokterdian@yahoo.com; saljunid@gmail.com<br>[*]corresponding author: Kampus UNNES, Sekaran, Gunungpati, Semarang, Indonesia. Phone: +62-24-8508007
Contributions	3b	MA, AF, MR drafted the manuscript. All authors contributed to the development of selection criteria, risk of a bias assessment strategy, and data extraction criteria. MA and AF developed the search strategy, UB and RS provided statistical and methodological expertise. MR provided expertise in COVID-19 from the perspective of pulmonary medicine, while DW provided expertise in the perspective of clinical pathology. UB and SA contributed to interpretation of the data. All authors read, provided feedback, and approved the final manuscript.

Amendments	4	Protocol amendments provided in PROSPERO: https://www.crd.york.ac.uk/prospero/display_record.php?RecordID=186306 (3 versions of the protocol: 29 May 2020; 02 July 2020; 04 August 2020)
Support:		
Sources	5a	This work was supported by the Ministry of Research and Technology/ National Research and Innovation Agency, Republic of Indonesia (Grant no. 056/SP2H/LT/DRPM/2020)
Sponsor	5b	Ministry of Research and Technology/ National Research and Innovation Agency, Republic of Indonesia
Role of sponsor or funder	5c	Sponsor had no role in the study design, data collection, data analysis, data interpretation, or writing of the manuscript
**INTRODUCTION**		
Rationale	6	The World Health Organization (WHO) has provided criteria for assessing the recovery of patients hospitalized with coronavirus disease 2019 (COVID-19), i.e. generally after clinical recovery and two negative PCR swabs > 24 hours apart. Recently, there have been several reports of recurrent SARS-CoV-2 RNA positivity in individuals who had recovered from COVID-19. with estimates of the incidence of recurrent SARS-CoV-2 positivity in individuals who have recovered from COVID-19, ranging from 7.3% to 21.4%. However, to date no systematic reviews have been published to provide a pooled estimate of the incidence of recurrent positivity
Objectives	7	This systematic review aimed to: estimate the incidence of recurrent SARS-CoV-2 positivity and determine the characteristics and risk factors related to the recurrent SARS-CoV-2 positivity in patients who had recovered from COVID-19
**METHODS**		
Eligibility criteria	8	The eligibility criteria were: (i) the study designs are cross-sectional, case-control or cohort design; (ii) the study reports the incidence of recurrent SARS-CoV-2 positivity in individuals who had recovered from COVID-19 and its related factors; and (iii) the articles included published or unpublished studies. The published studies may included both peer-reviewed reports and pre-print reports. Studies in languages other than English were excluded if no translated version of the manuscript was available.
Information sources	9	PubMed, MedRxiv, BioRxiv, Cochrane Library, ClinicalTrials.gov, the WHO international register of clinical trials registry
Search strategy	10	The search term in Medical Subjects Headings (MeSH) and free text: ("2019 nCoV" OR "2019nCoV" OR "2019 novel coronavirus" OR "COVID 19" OR "COVID19" OR "new coronavirus" OR "novel coronavirus" OR "SARS CoV-2" OR (Wuhan AND coronavirus) OR "COVID 19" OR "SARS-CoV" OR "2019-nCoV" OR "SARS-CoV-2") AND ((recurrence) OR (relapse) OR (re*infection) OR (re*activation)).
Study records: Data management	11a	Literature search results were organized using Mendeley (Mendeley, Ltd, Elsevier, UK).

Selection process	11b	The eligibility of articles based on their title and abstract was assessed independently by MA and AF. If necessary, the full paper was retrieved to further determine the eligibility status. In cases of disagreement regarding eligibility, consensus was reached by consulting a third reviewer (MR).
Data collection process	11c	Article titles and abstracts retrieved from the databases were transferred to Mendeley citation manager after being screened and checked for duplication. All records that did not meet the eligibility criteria were excluded from the review
Data items	12	Extracted data invlolved: authors, funding, study design, the population of the study, number of episodes of recurrent SARS-CoV-2 positivity per case, and patient characteristics that included age, sex, body mass index, clinical/laboratory manifestations, and comorbidities such as diabetes and hypertension, as well as recurrent SARS-CoV-2 positivity status.
Outcomes and prioritization	13	The outcome was recurrent SARS-CoV-2 positivity in individuals who had recovered from COVID-19, determined as based on positive result of reverse transcription polymerase chain reaction (RT-PCR) on re-testing, after being followed-up or re-admitted after discharged from hospital
Risk of bias in individual studies	14	We used the quality assessment tool for cross-sectional and cohort studies published by the National Institutes of Health to assess the methodological quality of included studies and the risk of bias.
Data synthesis	15a	We performed data analysis using Revman (Review Manager version 5.3.5 Copenhagen, The Nordic Cochrane Centre, 2014). Random-effects meta-analysis was used to calculate the pooled incidence of recurrent SARS-CoV-2 positivity with 95% confidence intervals. The incidence for each individual study with its standard error (SE) adds to the study data in RevMan. If the SE was not reported and the raw data could not be accessed, the SE was calculated using the formula $SE = \sqrt{(p}(1-p)/n)$. Meta-analysis was used to calculate pooled estimates of the time from disease onset to recurrent test positivity and the time from the last negative test result to recurrent positivity.
	15b	Meta-analysis was also used to calculate the pooled relative risk (RR) of recurrent SARS-CoV-2 positivity according to age, sex, hypertension, diabetes, other co-morbidities, disease severity, body mass index (BMI), fever as the initial presenting compliant, days from onset to negative conversion, lymphocyte count, D-dimer, and lung consolidation. We then assessed the heterogeneity between studies using I^2, with values of 25%, 50%, and 75% representing low, moderate, and high heterogeneity, respectively
	15c	A sensitivity analysis was also performed for non-peer-reviewed as well as for the PCR test specimens' type
	15d	
Meta-bias(es)	16	Due to the insufficient study data, meta-regression could not be performed.
Confidence in cumulative evidence	17	We measured the risk of bias using the National Institutes of Health's quality assessment tool

*** It is strongly recommended that this checklist be read in conjunction with the PRISMA-P Explanation and Elaboration (cite when available) for important clarification on the items. Amendments to a review protocol should be tracked and dated.** **The copyright for PRISMA-P (including checklist) is held by the PRISMA-P Group and is distributed under a Creative Commons Attribution Licence 4.0.**

From: Shamseer L, Moher D, Clarke M, Ghersi D, Liberati A, Petticrew M, Shekelle P, Stewart L, PRISMA-P Group. Preferred reporting items for systematic review and meta-analysis protocols (PRISMA-P) 2015: elaboration and explanation. BMJ. 2015 Jan 2;349(jan02 1):g7647.

Supplementary Table 2. Risk of bias assessment of Recurrent SARS-CoV-2 RNA positivity after COVID-19: A systematic review and meta analysis

		Study													
	Assessment	An, Jianghong	Chen	Huang	Hui Zhu	Jiang	Li	Liu	Wong	Xiao	Ye	Yuan	Zheng	Ling	Wu
1	1. Was the research question or objective in this paper clearly stated?	NA	Yes	Yes	Yes	No	Yes	No	Yes	Yes	Yes	Yes	Yes	Yes	Yes
2	2. Was the study population clearly specified and defined?	Yes	Yes	Yes	Yes	Yes	Yes	No	Yes	Yes	Yes	Yes	Yes	Yes	Yes
3	3. Was the participation rate of eligible persons at least 50%?	Yes	Yes	Yes	Yes	No	Yes	Yes	Yes	Yes	Yes	Yes	Yes	Yes	Yes
4	4. Were all the subjects selected or recruited from the same or similar populations (including the same time period)? Were inclusion and exclusion criteria for being in the study prespecified and applied uniformly to all participants?	Yes	Yes	Yes	Yes	Yes	Yes	Yes	Yes	Yes	Yes	Yes	Yes	Yes	Yes
5	5. Was a sample size justification, power description, or variance and effect estimates provided?	No	No	No	No	No	No	No	No	No	No	No	No	No	No
6	6. For the analyses in this paper, were the exposure(s) of interest measured prior to the outcome(s) being measured?	Yes	Yes	Yes	Yes	Yes	Yes	Yes	Yes	Yes	Yes	Yes	No	Yes	Yes
7	7. Was the timeframe sufficient so that one could reasonably expect to see an association between exposure and outcome if it existed?	Yes	Yes	Yes	Yes	Yes	Yes	Yes	Yes	Yes	Yes	Yes	No	No	Yes
8	8. For exposures that can vary in amount or level, did the study examine different levels of the exposure as related to the outcome (e.g., categories of exposure, or exposure measured as continuous variable)?	Yes	Yes	Yes	Yes	Yes	Yes	No	Yes	No	Yes	Yes	No	NA	NA
9	9. Were the exposure measures (independent variables) clearly defined, valid, reliable, and implemented consistently across all study participants?	Yes	Yes	Yes	Yes	Yes	Yes	Yes	Yes	No	Yes	Yes	Yes	Yes	Yes
10	10. Was the exposure(s) assessed more than once over time?	No	No	Yes	No	No	Yes	No	No	No	Yes	Yes	No	No	No
11	11. Were the outcome measures (dependent variables) clearly defined, valid, reliable, and implemented consistently across all study participants?	Yes	Yes	Yes	Yes	Yes	Yes	Yes	Yes	Yes	Yes	Yes	Yes	Yes	Yes
12	12. Were the outcome assessors blinded to the exposure status of participants?	NA	Yes	NA	NA	NA	NA	NA	NA	NA	No	No	NA	No	Yes
13	13. Was loss to follow-up after baseline 20% or less?	Yes	Yes	Yes	Yes	Yes	Yes	Yes	Yes	NA	Yes	Yes	No	NA	Yes
14	14. Were key potential confounding variables measured and adjusted statistically for their impact on the relationship between exposure(s) and outcome(s)?	Yes	No	Yes	Yes	No	No	NA	No	NA	No	No	NA	NA	Yes
	**Total Score**	10	11	12	11	8	11	7	10	7	11	11	6	7	11
	**Category of RoB**	Medium RoB	Low RoB	Low RoB	Low RoB	Medium RoB	Low RoB	Medium RoB	Medium RoB	Medium RoB	Low RoB	Low RoB	High RoB	Medium RoB	Low RoB

RoB: Risk of Bias

Low	0-6
Medium	7 -- 11
High	11>

Choice: Yes: clearly stated, No: not stated, NA: unclear

Supplementary Table 3. Sensitivity analysis summary of the non-peer-reviewed study and PCR test specimens' type for the meta-analysis of the pooled estimated incidence of recurrent SARS-CoV-2 RNA positivity

**Incidence**	Pool estimated incidence (%)	95%CI	I^2(%)
Total	14.8	11.44 – 18.19	78
Peer reviewed	14.6	11.05 – 18.09	75
**PCR test specimens type**			
Oropharyngeal alone	7.6	6.18 – 9.06	0
Nasopharyngeal alone	17.3	14.29 – 20.24	0
Oro-/naso-pharyngeal alone	13.5	8.95 – 17.95	85
Fecal alone	16.7	7.68 – 25.72	1 study
Combined (oro-/naso-pharyngeal and fecal)	16.1	12.04 – 20.06	22

PCR: PCR=rt-PCR: reverse transcription-polymerase chain reaction

Supplementary Table 4. Sensitivity analysis summary of the non-peer-reviewed study and PCR test specimens' type for the meta-analysis of the pooled estimated RR of age, sex, and BMI to recurrent SARS-CoV-2 RNA positivity

	Pool estimated RR/ mean difference	95%CI	I^2(%)
**Age**			
Total	**-2.4**	**-2.95 – -1.80**	99
Peer reviewed	1.6	0.93 – 2.31	93
**PCR test specimens type**			
Nasopharyngeal alone	**-8.6**	**-9.58 – -7.68**	99
Oro-/naso-pharyngeal alone	**-2.3**	**-2.87 – -1.72**	99
Combined (oro-/naso-pharyngeal and fecal)	**-6.5**	**-10.73 – -2.27**	1 study
**Sex**			
Total	0.8	0.67 – 1.05	0
Peer reviewed	0.9	0.71 – 1.21	0
**PCR test specimens type**			
Nasopharyngeal alone	**0.7**	**0.49 – 0.99**	0
Oro-/naso-pharyngeal alone	0.8	0.65 – 1.07	0
Combined (oro-/naso-pharyngeal and fecal)	0.8	0.50 – 1.40	0
**BMI**			
Total	0.2	-0.25 – 0.68	97
Peer reviewed	-0.4	-0.91 – 0.11	1 study
**PCR test specimens type**			
Nasopharyngeal alone	0.2	-0.25 – 0.68	97
Oro-/naso-pharyngeal alone	0.2	-0.25 – 0.68	97
Combined (oro-/naso-pharyngeal and fecal)	-	-	-

BMI: body mass index; PCR: PCR=rt-PCR: reverse transcription-polymerase chain reaction

Supplementary Table 5. Sensitivity analysis summary of the non-peer-reviewed studies and PCR test specimens' type for the meta-analysis of the pooled estimated RR of comorbidity, hypertension, and DM to recurrent SARS-CoV-2 RNA positivity

	Pool estimated RR	95%CI	I^2(%)
**Comorbidity**			
Total	0.9	0.64 – 1.29	52
Peer reviewed	1.2	0.79 – 1.70	2
**PCR test specimens type**			
Nasopharyngeal alone	**0.4**	**0.18 – 0.92**	0
Oro-/naso-pharyngeal alone	0.9	0.61– 1.28	63
Combined (oro-/naso-pharyngeal and fecal)	1.1	0.42 – 3.06	64
**Hypertension**			
Total	0.9	0.53 – 1.60	45
Peer reviewed	1.1	0.35 – 3.19	61
**PCR test specimens type**			
Nasopharyngeal alone	0.9	0.56 – 1.49	0
Oro-/naso-pharyngeal alone	0.8	0.58 – 1.15	0
Combined (oro-/naso-pharyngeal and fecal)	6.8	0.98 – 46.62	1 study
**Diabetes mellitus**			
Total	**0.5**	**0.30 – 0.90**	53
Peer reviewed	0.8	0.45 – 1.57	0
**PCR test specimens type**			
Nasopharyngeal alone	**0.3**	**0.11 – 0.72**	0
Oro-/naso-pharyngeal alone	**0.5**	**0.30 – 0.90**	53
Combined (oro-/naso-pharyngeal and fecal)	-	-	-

PCR: PCR=rt-PCR: reverse transcription-polymerase chain reaction

Supplementary Table 6. Sensitivity analysis summary of the non-peer-reviewed study and PCR test specimens' type for the meta-analysis of the pooled estimated RR of fever and clinical features to recurrent SARS-CoV-2 RNA positivity

	Pool estimated RR	95%CI	I^2(%)
**Fever**			
Total	1.0	0.76 – 1.32	0
Peer reviewed	1.1	0.77 – 1.57	0
**PCR test specimens type**			
Nasopharyngeal alone	0.8	0.56 – 1.22	0
Oro-/naso-pharyngeal alone	0.9	0.64 – 1.20	0
Combined (oro-/naso-pharyngeal and fecal)	1.6	0.85 -2.82	0
**Severity**			
Total	**0.5**	**0.35 – 0.84**	70
Peer reviewed	0.9	0.51 – 1.48	6
**PCR test specimens type**			
Nasopharyngeal alone	**0.2**	**0.10 – 0.58**	1 study
Oro-/naso-pharyngeal alone	**0.6**	**0.37 – 0.92**	77
Combined (oro-/naso-pharyngeal and fecal)	0.2	0.01 – 2.36	1 study
**Consolidation**			
Total	1.2	0.87 – 1.66	58
Peer reviewed	1.5	0.98 – 2.18	48
**PCR test specimens type**			
Nasopharyngeal alone	0.8	0.45 – 1.28	0
Oro-/naso-pharyngeal alone	1.2	0.87 – 1.66	58
Combined (oro-/naso-pharyngeal and fecal)	-	-	-
**Lymphocyte count <1.1**			
Total	**0.6**	**0.39 – 0.86**	48
Peer reviewed	0.7	0.39 – 1.16	58
**PCR test specimens type**			
Nasopharyngeal alone	**0.5**	**0.30 – 0.83**	0
Oro-/naso-pharyngeal alone	**0.5**	**0.36 – 0.82**	0
Combined (oro-/naso-pharyngeal and fecal)	3.3	0.76 – 14.58	1 study
**d-Dimer <0.5**			
Total	1.3	0.97 – 1.97	0
Peer reviewed	1.3	0.79 – 2.20	0
**PCR test specimens type**			
Nasopharyngeal alone	1.4	0.80 – 2.49	0
Oro-/naso-pharyngeal alone	1.3	0.97 – 1.97	0
Combined (oro-/naso-pharyngeal and fecal)	-	-	-

PCR: PCR=rt-PCR: reverse transcription-polymerase chain reaction
